# Influence of polymorphisms in candidate genes on carcass and meat quality traits in rabbits

**DOI:** 10.1371/journal.pone.0294051

**Published:** 2023-11-09

**Authors:** Hosam M. Safaa, Mohamed Ragab, Marwa Ahmed, Belal El-Gammal, Mostafa Helal

**Affiliations:** 1 Department of Biology, College of Science, University of Bisha, Bishah, Saudi Arabia; 2 Animal Production Department, Faculty of Agriculture, Cairo University, Giza, Egypt; 3 Poultry Production Department, Faculty of Agriculture, Kafrelsheikh University, Kafrelsheikh, Egypt; 4 Animal Breeding and Genetics Department, National Institute for Agricultural and Food Research and Technology (INIA), Madrid, Spain; 5 Department of Animal Production, National Research Center, Dokki, Giza, Egypt; 6 Department of Chemistry, College of Science, University of Bisha, Bishah, Saudi Arabia; Ain Shams University Faculty of Agriculture, EGYPT

## Abstract

**C**andidate gene is a powerful approach to study gene-trait association and offers valuable information for genetic improvement using marker-assisted selection. The current work aimed to study the polymorphisms of four single nucleotide polymorphism (SNPs) at located growth hormone (*GH*), insulin-like growth factor-II (*IGF-II*), fat mass and obesity-associated (*FTO*), and insulin receptor substrate-1 (*IRS-1*) genes, and their association with the carcass, and meat quality traits in rabbits. The SNPs were genotyped using RFLP-PCR in New Zealand White and local Baladi rabbits. The results revealed that the heterozygous genotype was the most frequent in all cases, except for the *FTO* SNP in LB rabbits. There was a significant effect for *GH* genotypes on meat lightness after slaughter and hind-part weight. While, *IGF-II* mutation significantly affected slaughter, hot carcass, commercial carcass, and hind-part weights. The *FTO* SNP was associated with cooking loss and intramuscular fat weight, and the *IRS-1* SNP was significantly associated with drip loss and intramuscular fat. Specific-breed effects were obtained for *IGF-II* SNP on cooking loss, and for the intramuscular fat. Although the results suggested that these mutations are useful candidate genes for selection, more research for detecting more variants associated with carcass and meat quality traits in rabbits are recommended.

## 1. Introduction

Rabbits (*Oryctolagus cuniculus*) are prolific animals that help fill the gap between the production and consumption of animal protein [1]. However, commercial breeds are highly sensitive to high ambient temperatures, while local breeds are less sensitive to the local environmental condition [2]. Therefore, designing breeding programs to improve the performance of highly adapted local breeds is recommended [3]. The classical breeding methods such as mass selection were successful, and resulted in improved breeds and lines [4]. However, the classical breeding approaches are not suitable for some characteristics such as carcass and meat quality traits [5], where the phenotypic data for those traits are collected after slaughtering the animals, which reduces the effectiveness of selective breeding due to the use of indirect and family selection [6].

The approaches of molecular genetics have facilitated and accelerated the genetic improvement process, with higher accuracies and shorter times [5]. Among these approaches is marker-assisted selection (MAS), where selection is practiced based on the combination of phenotypic and genotypic data [7]. Single nucleotide polymorphism (SNP) associated with a specific character is considered an important genetic marker for the application of MAS. Additionally, the associations between variants and traits are economically important, as the association lies in their potential to improve different traits [8]. Also, the identification of the genetic variations associated with a trait allows for a better understanding of the underlying biological mechanisms and pathways that contribute to the trait, which can be used to improve these traits to increase the productivity [9, 10]. Nevertheless, the information about the association between SNPs in different candidate genes and economic traits, in rabbits, are is still limited [11–14].

Like other species, growth hormone (GH) in rabbits is essential for growth and development. The GH hormone is the product of the *GH* gene (ENSOCUT00000004342.4), which is located on chromosome 19 in rabbits, and comprises 4 introns and 5 exons, coding for a protein of 216 amino acids and 190 amino acids mature peptides [15]. The GH in rabbits has a vital role in postnatal growth and is involved in the regulation of different biological and metabolic functions, including muscle mass accretion, and bone growth [16–18]. The polymorphisms in the *GH* gene have shown significant associations with body weight in rabbits [15, 19]. However, there is only one study that addressed the association of *GH* polymorphisms with carcass or meat quality traits in rabbits [20].

Insulin-like growth factor 2 (IGF-2) is the product of the *IGF-II* gene (NM_001171406) that is located on chromosome 4 in rabbits. It has a function similar to insulin and IGF-1 hormones, as it involves mediating growth and development, and promotes the growth of different tissue by binding to the IGFs receptor [21]. Both IGF-1 and IGF-2 have autocrine and paracrine actions. The polymorphisms of the *IGF-II* gene have been associated with different traits in different animals, such as body weights in rabbits [15, 22], backfat thickness in pigs [23], growth and meat production in pigs [24], and milk production in cattle [25].

The fat mass and obesity-associated (FTO) protein also play an essential role in postnatal growth. The *FTO* gene (ENSOCUG00000001741) is located on chromosome 5 in rabbits and encodes the FTO protein. Loss of the *FTO* function leads to postnatal growth retardation with a significant reduction in adipose tissue, and lean body mass [26]. Rabbit *FTO* shared 36.36–91.88% identity with the *FTO* gene from other species, and is also closely related to humans [27]. Studies exploring the polymorphism of the *FTO* gene in rabbits are scarce. Only one study investigated the mutations of the *FTO* gene in rabbits and reported a missense mutation (c.499G>A, p.A167T) associated with body weight in New Zealand rabbits [28]. Nevertheless, studies on different species suggested that *FTO* intron 1 SNPs may be associated with increased energy intake [29, 30].

The insulin receptor substrate 1 (*IRS*-*1*) gene is another important gene that is associated with the previous ones [31, 32]. The *IRS-1* gene in rabbits is located on chromosome 7 (ENSOCUG00000007187) [33]. This gene plays a significant role in signal transduction of IGFs and GH signaling pathways, and it has roles in postnatal growth and in lipid metabolism [34]. In rabbits, Zhang et al. [35] sequenced the *IRS-1* gene and detected nine synonymous mutations, and only two SNPs (c.189G>T and c.2574G>A) were associated with growth traits. Further analysis for the c.189G>T mutation was performed [9] and confirmed the association with growth traits. Variants of the *IRS-1* gene were also associated with growth in sheep [36].

In recent years, the candidate-gene approach has proven effective in identifying several SNPs associated with meat rabbits [37–39]. However, the identification of candidate genes for carcass and meat quality traits in rabbits has been scarce [33, 39]. Therefore, the present study endeavors to identify novel candidate SNPs that are linked to these particular traits of carcass and meat quality in rabbits, this includes evaluating the association between four SNPs located at *GH* (c.-78C>T), *IGF-2* (c.156+61delA), *FTO* (c.499G> A), and *IRS-1* (c.189G>T) genes with carcass and meat quality traits in two rabbit breeds.

## 2. Materials and methods

### 2.1. Ethical approval

All procedures of the current study were approved from the institutional animal care and use committee at Cairo University (CU-IACUC), approval number is CU-IACUC/ I /F/46/22.

### 2.2. Animals

In the present study, data from 120 rabbits were analyzed: 71 (33 ♂♂ and 38 ♀♀) were New Zealand White (NZW) rabbits as a commercial line, and 49 (25 ♂♂ and 24 ♀♀) were improved local Baladi rabbits (LB). Rabbits were collected from different places and sources based on their age (5 weeks of age) and body weights (788±34 g for NZW, and 645±41g for local Baladi rabbits). All rabbits were housed under the same conditions (summer season) during the fattening period in a semi-closed housing system, ambient temperatures ranged from 17 (low) to 33 (high) °C with a light period of about 16h/day, and relative humidity ranged from 40 to 60%. Rabbits were raised in grouping cages (60 × 50 × 40 cm) and received the same management practices, where they were fed *ad libitum* commercial pellets fattening diet (18% protein, 2700 Kcal metabolic energy, and 12% crude fiber per Kg), and water was provided *ad libitum* via an automatic nipple system.

### 2.3. Studied traits

At marketing age (12 weeks), rabbits were slaughtered after 12 hours of fasting with free access to water. Rabbits were anesthetized by intravenous injection of Ketamine (10 mg/kg) and Xylazine (3 mg/ kg), followed by exsanguination. Carcasses were then dissected according to that previously reported [40]. The recorded traits included slaughter body weight (SW, g), hot carcass weight 15 min after slaughter (HCW, g), commercial carcass weight 24h after slaughter at -4°C (CCW, g), hind parts weight (HPW, g). Drip loss (DL, %) was calculated as the difference between HCW and CCW divided by HCW (×100). Dressing out percentage (DOP, %) was calculated by dividing CCW by SW (×100). Hind parts (HPW, g), liver, and kindly weights (%) were obtained as relative parentages to CCW.

Meat quality traits included measuring the pH of the muscles two times (0 h and 24 h after slaughtering) on the *Longissimus dorsi* muscle carcass surface. Meat color was also determined two times (0 h and 24 h after slaughtering) using Chroma meter (Konica Minolta, Japan) at different locations of the *Longissimus dorsi* muscle, and expressed by lightness (L*), redness (a*), and yellowness (b*) color system. Cooking loss was calculated according to Pascual and Pla [41].

### 2.4. Genotyping

Individual blood samples were collected from rabbits before slaughter. DNA was extracted using WizPrep genomic DNA kit (wiz-biosolution Inc., South Korea). Polymerase chain reaction (PCR) and amplifications of the different gene sequences were performed using a thermal cycler machine (Techne, UK). The PCR reaction was adjusted to 20 μl, including 2 μl of DNA template, 1 μl of each of the forward and reverse primers, 8 μl of mater mix, and 8 μl of nuclease-free water. The PCR program included the initial denaturation step (94°C for 5 min), 35 cycles of amplification (denaturation at 94°C for 1 min, annealing at 53–57°C for 45 sec, and elongation at 72°C for 45 sec), and the final elongation step was set to 72°C for 5 min. The primers were designed to amplify fragments that included one mutation in each of the *GH* (Part of the 5’-flanking region and 5’-untranslated region, exon 1), *IGF-2* (136 bp, part of the first coding exon, and part of the downstream intron), *FTO* (233 bp of exon 3), and *IRS1* (CDs and 5’-untranslated region of exon 1) genes. The mutations were then genotyped using the restriction fragment length polymorphism (RFLP) technique as shown in Table 1. The primers and restriction enzymes were described previously for the c.-78C>T mutation of *GH* [17], *IGF2* [15, 22], and *IRS-1* genes [35]. For the *FTO* gene mutation, primers were designed using Primer Designing Tool of NCBI (https://www.ncbi.nlm.nih.gov/tools/primer-blast/) to amplify a fragment of 233 bp of exon 3 that was previously sequenced [28].

**Table 1:** Primer sequences, fragment size, and genotyping method for the genotyped mutations.

Gene	Genotyped mutation	Primers	Restriction Enzyme	Fragment size (bp)	References
*GH*	c.-78C>T	GTATAGTGGGATGGGGTTGG TTACGCTCCCATTCAGAAGC	*Bsh1236I*	T = 231 C = 169, 62	(Fontanesi et al. 2012a)
*IGF2*	c.156+61delA	GGACACCCTCCAGTTTGTGT CAGCAGGTGTTCCGCAAG	*HpyF31 (Ddel)*	A = 103, 33 Del = 136	(Fontanesi et al. 2012b)
*FTO*	c.499G > A	TGGCCCGTGAAAGGTTCTAC TGTCCACTTCATCTTGTCCATCA	*Hpy188I*	A = 223 G = 127 +96	(Zhang et al. 2013)
*IRS1*	c.189G >T	GGCGGCGTGGATTTCAGA CGCAATGGCAAAGTGTTCG	*Sau3AI*	G = 432 T = 332‏, 100	(Zhang et al. 2014)

### 2.5. Statistical analysis

The association analysis between the different mutations and studied traits was performed using General Linear Model (GLM) procedure of SAS [42]. The following model was used for the analysis:

$$Y_{ijklm}=m+B_{i}+S_{j}+L_{k}+G_{l}+e_{ijklm}$$

The model included the fixed effects of breed (*B*_*i*_), sex (*S*_*j*_), litter size (*L*_*k*_), and genotype (*G*_*l*_). Hardy-Weinberg equilibrium of the different SNPs was tested using χ^2^. Differences with *p*-value less than 0.05 were considered statistically significant. The additive and dominance effects were calculated according to Russo et al. [43].

## 3. Results

The descriptive statistics of the studied traits are presented in Table 2. The NZW rabbits were significantly higher than Baladi rabbits in SW, HCW, and CCW traits. The drip loss was also higher in NZW compared to Baladi rabbits. While, the other traits were not significantly differed between the two genetic groups, including pH0, pH24, meat color after slaughtering (L*^0^, a*^0^, and b*^0^), meat color after 24 hours of slaughtering (L*^24^, a*^24^, and b*^24^), CL, IMF, DOP, and HPW traits.

**Table 2:** Means (+SD) of the different traits in the two rabbit breeds.

Trait	NZW rabbits	Baladi rabbits	*p-value*
**pH after slaughtering, pH0**	6.68+0.012	6.66+0.009	0.2320
**pH 24 h after slaughtering, pH24**	5.766+0.011	5.758+0.01	0.5840
Redness (a*) after slaughtering, a* ^**0**^	4.3+0.003	4.294+0.004	0.2610
Redness (a*) 24 h after slaughtering, a* ^**24**^	4.802+0.005	4.806+0.006	0.5636
Lightness (L*) after slaughtering, L* ^**0**^	50.325+0.007	50.327+0.007	0.8179
Lightness (L*) 24 h after slaughtering, L* ^**24**^	58.344+0.021	58.353+0.022	0.7733
Yellowness (b*) after slaughtering, b* ^**0**^	3.312+0.002	3.312+0.003	0.9717
Yellowness (b*) 24 h after slaughtering, b* ^**24**^	5.532+0.016	5.521+0.005	0.5867
**Cooking loss, CL (%)**	28.295+0.329	28.674+0.433	0.4801
**Intramuscular fat, IMF (g)**	0.435+0.008	0.416+0.008	0.1107
**Slaughter weight, SW (g)**	2548.480^a^+107.1	2295.66^b^+148.01	0.0001
**Hot carcass weight, HCW (g)**	1563.940^a^+81.4	1398.12^b^+128.7	0.0001
**Commercial carcass weight, CCW (g)**	1491.34^a^+79.54	1342.10^b^+124.22	0.0001
**Drip loss, DL (%)**	4.571^a^+0.076	4.029^b^+0.093	0.0001
**Dressing out percentage, DOP (%)**	59.336+0.333	58.44+0.307	0.0618
**Liver (%)**	5.528+0.046	5.538+0.046	0.8787
**Kidney (%)**	0.988+0.005	0.985+0.004	0.6974
**Hind part weight, HPW (%)**	38.64+0.381	37.595+0.366	0.0602

The allelic and genotypic frequencies of the different SNPs in the two rabbit breeds are presented in Table 3. All the targeted alleles were represented in the two populations, and the heterozygous genotype was the most frequent in all cases and ranged between 0.35 to 0.48, except for the SNP c.499G > A of the *FTO* gene in LB rabbits. For the *GH* gene, the allelic frequencies differed between the breeds, where allele T was the predominant allele in NZW rabbits. While in LB rabbits, allele C was the predominant. A similar situation was observed for *IGF-2* mutation. However, the G and G alleles were predominant in the two breeds for the mutations of *FTO* and *IRS-1* genes, respectively. Table 3 also shows the exacted heterozygosity (He) levels, which ranged from 0.497 to 0.499 for *GH* mutation, 0.484 to 0.500 for *IGF-2* mutation, 0.490 to 0.493 for *FTO* mutation, and 0.480 to 0.495 for *IRS-1* mutation. The number of effective alleles was relatively high and ranged from 2.000 to 2.083 for all mutations in the two breeds, and the polymorphic information content ranged from 0.367 to 0.375. All frequency distributions were consistent with Hardy-Weinberg’s law.

**Table 3:** Genotypic frequency, allelic-frequency and deviation from Hardy Weinberg of the different mutations.

Breed	Genotypic frequency			Allelic frequency		Genetic parameters			Deviation form HW	
	*p2*	*2pq*	*q2*	*p*	*q*	*He*	*Ne*	*PIC*	*χ* ^*2*^	*p-value*
	*GH (c*.*-78C>T)*									
NZW	0.23	0.48	0.30	0.46	0.54	0.497	2.012	0.373	0.099	0.951
LB	0.31	0.41	0.29	0.51	0.49	0.499	2.004	0.375	1.646	0.439
All breeds	0.26	0.45	0.29	0.48	0.52	0.499	2.004	0.375	1.176	0.055
	*IGF-2 (c*.*156+61delA)*									
NZW	0.25	0.39	0.35	0.45	0.55	0.495	2.020	0.373	2.941	0.23
LB	0.39	0.41	0.20	0.59	0.41	0.484	2.066	0.367	1.179	0.554
All breeds	0.31	0.38	0.32	0.50	0.50	0.500	2.000	0.375	7.499	0.024
	*FTO (c*.*499G > A)*									
NZW	0.34	0.45	0.21	0.56	0.44	0.493	2.028	0.371	0.399	0.799
LB	0.41	0.35	0.24	0.58	0.42	0.490	2.041	0.369	3.039	0.133
All breeds	0.37	0.41	0.23	0.57	0.43	0.490	2.041	0.370	3.331	0.189
	*IRS-1 (c*.*189G>T)*									
NZW	0.34	0.45	0.21	0.56	0.44	0.493	2.028	0.371	0.499	0.779
LB	0.35	0.37	0.29	0.53	0.47	0.480	2.083	0.374	3.378	0.185
All breeds	0.34	0.42	0.24	0.55	0.45	0.495	2.020	0.373	3.005	0.226

NZW = New Zealand rabbits, LB = Local Baladi rabbits, AA = Dominant homozygous genotype, Aa = heterozygous genotype, aa = recessive homozygous genotype, He = expected heterozygosity, Ne = number of effective alleles, PIC = polymorphic information content. χ2 represents the balance of Hardy–Weinberg of different genotypic distributions: χ^2^_0.05_ = 3.84, χ^2^_0.01_ = 6.63.

Table 4 shows the association between *GH* mutation (c.-78C>T) and different traits in the two breeds. The obtained results indicated significant associations for the genotypes with the meat lightness after slaughtering (L*^0^, p < 0.04) and HPW (p < 0.001) in both breeds. The heterozygous genotype (TC) was superior to the homozygous genotype (CC) in the two breeds, for meat lightness after slaughtering. Regarding HPW, the trait was influenced by the mutation in the two breeds, where rabbits with TT genotype had significantly higher HPW than the two other genotypes. Also, there was a significant (p < 0.001) breed specific effect on yellowness after 24 hours of slaughtering (b*^24^) in LB rabbits, where the heterozygous genotype had significantly higher values of b*^24^ compared to the CC genotype, which was not observed in NZW rabbits. The results also denoted that the estimated dominant genetic effects of *GH* gene were higher than the additive genetic effects.

**Table 4:** The association analysis of the GH c.-78C>T polymorphism on carcass and meat quality traits in New Zealand White and local Baladi rabbits.

	New Zealand White			Additive	Dominance	*p-value*	Local Baladi			Additive	Dominance	*p-value*
	TT	TC	CC				TT	TC	CC			
**pH0**	6.69± 0.03	6.67± 0.02	6.69± 0.02	0.002	-0.021	0.7423	6.64± 0.01	6.69± 0.03	6.66± 0.01	-0.006	0.037	0.1804
**pH24**	5.80± 0.02	5.75± 0.02	5.76± 0.02	0.017	-0.034	0.2250	5.75± 0.02	5.78± 0.03	5.75± 0.01	-0.002	0.033	0.3545
a* ^**0**^	4.30± 0.01	4.32± 0.01	4.30± 0.01	0.000	0.007	0.6651	4.30± 0.01	4.30± 0.01	4.29± 0.01	0.003	0.008	0.5304
a* ^**24**^	4.79± 0.01	4.80± 0.01	4.81± 0.01	-0.007	0.005	0.4273	4.80± 0.01	4.82± 0.01	4.81± 0.01	-0.006	0.014	0.4134
L* ^**0**^	50.30^b^± 0.02	50.34^a^± 0.01	50.32^ab^± 0.01	-0.010	0.029	0.0431	50.31^b^± 0.02	50.35^a^± 0.01	50.32^ab^± 0.01	-0.005	0.034	0.0401
L* ^**24**^	58.38± 0.05	58.31± 0.03	58.35± 0.03	0.014	-0.049	0.5763	58.33± 0.02	58.35± 0.05	58.38± 0.04	-0.024	-0.007	0.6434
b* ^**0**^	3.31± 0.01	3.31± 0.01	3.31± 0.00	-0.001	-0.005	0.6350	3.31± 0.01	3.31± 0.01	3.31± 0.01	-0.001	0.001	0.9771
B* ^**24**^	5.52± 0.01	5.52± 0.01	5.55± 0.03	-0.016	-0.015	0.6235	5.50^b^± 0.01	5.53^a^± 0.01	5.53^a^± 0.01	-0.015	0.011	0.0098
**CL**	28.36± 0.79	28.197± 0.642	28.32± 0.44	0.021	-0.146	0.981	28.35± 0.94	29.70± 0.73	28.20± 0.60	0.078	1.422	0.3311
**IMF**	0.412± 0.02	0.449± 0.01	0.44± 0.01	-0.013	0.025	0.2205	0.44± 0.02	0.41± 0.02	0.41± 0.01	0.016	-0.014	0.2393
**SW**	2542.58± 23.23	2541.26± 12.95	2555.72± 18.08	-6.57	-7.89	0.8177	2279.71± 19.93	2288.58± 12.74	2312.57± 32.12	-16.43	-7.56	0.6305
**HCW**	1559.89± 22.41	1571.99± 12.57	1560.87± 11.19	-0.490	11.610	0.8731	1376.88± 17.30	1384.79± 23.01	1423.37± 23.41	-23.24	-15.34	0.2625
**CCW**	1486.37± 21.80	1501.92± 12.56	1487.13± 10.80	-0.38	15.17	0.6950	1322.34± 17.07	1331.8± 22.17	1364.12± 22.66	-20.89	-11.43	0.3308
**DL**	4.53± 0.15	4.46± 0.17	4.66± 0.10	-0.064	-0.134	0.5264	4.07± 0.16	3.83± 0.07	4.14± 0.19	-0.032	-0.279	0.3814
**DOP**	59.30± 0.81	60.83± 0.65	58.43± 0.36	0.439	1.968	0.0068	58.000± 0.50	58.16± 0.75	58.97± 0.39	-0.484	-0.319	0.3649
**liver**	5.65± 0.10	5.49± 0.09	5.49± 0.07	0.0815	-0.08	0.3463	5.51± 0.09	5.48± 0.07	5.60± 0.08	-0.046	-0.077	0.5157
**Kidney**	0.98± 0.01	0.99± 0.01	0.90± 0.01	-0.001	0.007	0.8497	0.98± 0.01	0.99± 0.01	0.98± 0.01	-0.002	0.009	0.5661
**HPW**	35.54^c^± 0.42	37.71^b^± 0.49	40.68^a^± 0.49	-2.569	-0.401	0.0001	35.51c± 0.36	37.13b± 0.58	39.49a± 0.48	-1.988	-0.365	0.0001

pH0 & pH24 = pH after slaughtering and after 24 h of slaughtering; L*^0^ & L*^24^ = lightness (L*) after slaughtering and 24h after slaughtering; a*^0^ & a*^24^ = redness (a*) after slaughtering and 24h after slaughtering, b*^0^ & b*^24^ = yellowness (b*) after slaughtering and 24h after slaughtering; CL = cooking loss; IMF = intramuscular fat; SW = slaughter weight; HCW = hot carcass weight; CCW = commercial carcass weight; DL = Drip loss; DOP = dressing out percentage; HPW = hind part weight.

The associations between the mutation of the *IGF-2* gene and different traits in the two breeds are presented in Table 5. There was a significant effect of the mutation on the weight of the hind part in the two breeds (p < 0.0001 and 0.02 for NZW and LB, respectively), where rabbits with Del/Del genotype showed higher HPW compared with AA and A/Del genotypes. While in local rabbits, the deletion mutation had also effects on CL and IMF, as the mutant genotype had lower values compared to the other genotypes. The genetic dominance effects were also higher than the additive effect.

**Table 5:** The association analysis of the *IGF-2* c.156+61delA polymorphism on carcass and meat quality traits in New Zealand White and local Baladi rabbits.

	New Zealand White			Additive	Dominance	*p-value*	Local Baladi			Additive	Dominance	*p-value*
	AA	A/DEL	DEL/DEL				AA	A/DEL	DEL/DEL			
**pH0**	6.69± 0.03	6.70± 0.02	6.66± 0.01	0.013	0.022	0.4309	6.68± 0.02	6.66± 0.01	6.64± 0.01	0.018	-0.003	0.3435
**pH24**	5.76± 0.03	5.78± 0.02	5.76± 0.01	-0.005	0.019	0.7011	5.77± 0.02	5.75± 0.01	5.75± 0.01	0.0125	-0.013	0.5084
a* ^**0**^	4.31± 0.01	4.30± 0.01	4.30± 0.01	0.005	-0.008	0.3800	$\begin{array}{l} 4.29\pm 0.004 \end{array}$	4.30± 0.01	4.29± 0.01	-0.001	0.006	0.6876
a* ^**24**^	4.79± 0.01	4.82± 0.01	4.80± 0.01	-0.0001	0.021	0.1107	4.81± 0.01	4.80± 0.01	4.81± 0.01	0.004	-0.013	0.4332
L* ^**0**^	50.32± 0.01	50.32± 0.01	50.33± 0.01	-0.008	-0.003	0.6703	50.33± 0.01	50.33± 0.01	50.33± 0.01	-0.002	-0.006	0.9259
L* ^**24**^	58.366± 0.05	58.354± 0.04	58.32± 0.02	0.023	0.011	0.6733	58.39± 0.04	58.34± 0.03	58.32± 0.02	0.037	-0.016	0.3925
b* ^**0**^	3.30± 0.01	$\begin{array}{l} 3.32\pm 0.004 \end{array}$	3.32±0.004	-0.006	0.006	0.1016	3.31± 0.01	3.31± 0.00	3.31± 0.01	0.003	0.004	0.6478
b* ^**24**^	5.51± 0.01	5.52± 0.01	5.55± 0.04	-0.019	-0.009	0.6048	5.52± 0.01	5.538± 0.01	5.51± 0.01	0.005	0.014	0.3513
**CL**	29.01± 0.64	28.60± 0.51	27.57± 0.56	0.719	0.303	0.1857	28.59^ab^± 0.78	29.54^a^± 0.54	27.10^b^± 0.96	0.747	1.701	0.1118
**IMF**	0.44± 0.01	0.45± 0.01	0.45± 0.01	-0.004	-0.024	0.2968	0.43^a^± 0.01	0.43^a^± 0.01	0.38^b^± 0.02	0.025	0.023	0.0693
**SW**	2532.47± 20.63	2540.26± 18.78	2566.12± 16.79	-16.83	-9.035	0.4035	2294.79± 31.00	2281.16± 17.77	2326.28± 24.28	-15.75	-29.38	0.5407
**HCW**	1541.06± 17.64	1563.22± 14.56	1579.29± 10.97	-19.115	3.045	0.1833	1386.46± 24.83	1390.45± 18.10	1435.59± 20.08	-24.56	-20.58	0.3411
**CCW**	1468.61± 17.65	1490.90± 14.26	1506.33± 10.38	-18.86	3.43	0.1774	1331.38± 24.09	1335.12± 17.40	1376.41± 19.59	-22.52	-18.78	0.3806
**DL**	4.71± 0.13	4.42± 0.15	4.62± 0.11	0.0747	-0.244	0.3102	4.09± 0.11	3.95± 0.12	4.07± 0.34	0.012	-0.134	0.7727
**DOP**	59.17± 0.83	59.38± 0.53	59.41± 0.48	-0.122	0.089	0.9567	57.99± 0.55	58.51± 0.50	59.16± 0.44	-0.585	-0.063	0.3814
**liver**	5.56± 0.09	5.55± 0.07	5.46± 0.08	0.067	0.024	0.4907	5.53± 0.07	5.61± 0.07	5.42± 0.11	0.052	0.132	0.3387
**Kidney**	0.98± 0.01	1.00± 0.01	0.98± 0.01	0.003	0.019	0.2005	0.99± 0.01	0.98± 0.01	0.98± 0.01	0.006	-0.005	0.3862
**HPW**	36.60^b^± 0.51	37.85^b^± 0.49	40.66^a^± 0.63	-2.027	-0.785	0.0001	37.01^b^± 0.42	37.18^b^± 0.53	39.56^a^± 1.06	-1.275	-1.106	0.0215

pH0 & pH24 = pH after slaughtering and 24h after slaughtering; L*^0^ & L*^24^ = Lightness (L*) after slaughtering and 24h after slaughtering; a*^0^ & a*^24^ = redness (a*) after slaughtering and 24h after slaughtering, b*^0^ & b*^24^ = yellowness (b*) after slaughtering and 24h after slaughtering; CL = cooking loss; IMF = intramuscular fat; SW = slaughter weight; HCW = hot carcass weight; CCW = commercial carcass weight; DL = Drip loss; DOP = dressing out percentage; HPW = hind part weight.

Table 6 presents the associations of the mutation of the *FTO* gene with carcass and meat quality traits in the two breeds. There was a significant effect of the SNP in the rabbits of the two breeds on CL (p < 0.0001), IMF (p < 0.011), and HPW (p < 0.001), where the AA genotype in both breeds had higher CL and IMF, and lower HPW than the two other genotypes (GG and AG). In NZW rabbits, the mutant genotype (GG) had significantly lower (p < 0.0002) DL than the other genotypes. In local Baladi rabbits, the AG genotype had a significantly higher meat lightness value after slaughtering (L*^0^, p < 0.0006) and b*^0^ (p < 0.0007) than the AA genotypes. As noted for the *GH* gene, the estimated dominant genetic effects were higher than the additive genetic effects.

**Table 6:** The association analysis, by breed, of the *FTO* c.499G>A polymorphism on carcass and meat quality traits New Zealand White and local Baladi rabbits.

	New Zealand White			Additive	Dominance	*p-value*	Local Baladi			Additive	Dominance	*p-value*
	AA	AG	GG				AA	AG	GG			
**pH0**	6.69± 0.02	6.67± 0.02	6.68± 0.02	0.002	-0.012	0.8904	6.66± 0.01	6.66± 0.01	6.67± 0.031	-0.007	-0.002	0.8163
**pH24**	5.77± 0.02	5.78± 0.02	5.76± 0.02	0.006	0.013	0.7671	5.75± 0.01	5.75± 0.01	5.78± 0.03	-0.014	-0.010	0.5314
a* ^**0**^	4.29± 0.01	4.30± 0.01	4.31± 0.01	-0.006	0.002	0.3406	4.30± 0.01	4.30± 0.02	4.29± 0.01	0.006	0.009	0.3096
a* ^**24**^	4.80± 0.01	4.80± 0.01	4.81± 0.01	-0.002	-0.006	0.7052	4.81± 0.01	4.80± 0.01	4.81± 0.01	0.001	-0.012	0.5817
L* ^**0**^	50.32± 0.01	50.33± 0.012	50.33± 0.01	-0.005	0.001	0.8279	50.35^a^± 0.01	50.30^b^± 0.01	50.33^ab^± 0.01	0.005	-0.037	0.0063
L* ^**24**^	58.34± 0.04	58.35± 0.04	58.35± 0.04	-0.003	0.002	0.9936	58.34± 0.03	58.32± 0.01	58.42± 0.07	-0.037	-0.061	0.2258
b* ^**0**^	3.31± 0.01	3.32± 0.01	3.31± 0.01	0.000	0.007	0.3580	3.32^a^± 0.00	3.30^b^± 0.01	3.31^b^± 0.01	0.008	-0.011	0.0077
b* ^**24**^	5.52± 0.01	5.56± 0.05	5.51± 0.01	0.005	0.044	0.4263	5.52± 0.01	5.52± 0.01	5.52± 0.01	-0.002	0.002	0.8676
**CL**	29.80^a^± 0.26	29.62^a^± 0.42	25.69^b^± 0.51	2.053	1.873	0.0001	30.23^a^± 0.63	30.02^a^± 0.36	24.17^b^± 0.77	3.032	2.823	0.0001
**IMF**	0.47^a^± 0.01	0.43^b^± 0.01	0.41^b^± 0.0227	0.028	-0.011	0.0075	0.43^a^± 0.02	0.43^a^± 0.01	0.37^b^± 0.02	0.030	0.024	0.0117
**SW**	2546.87± 15.41	2561.91± 18.76	2538.22± 21.21	4.325	19.365	0.6705	2326.49± 30.24	2279.13± 17.59	2267.68± 19.55	29.405	-17.955	0.2176
**HCW**	1568.46± 11.89	1563.07± 12.535	1560.36± 17.22	4.050	-1.340	0.9179	1412.11± 25.63	1395.03± 19.68	1379.16± 14.10	16.475	-0.605	0.6061
**CCW**	1494.06± 11.51	1487.4± 12.30	1492.19± 16.88	0.935	-5.725	0.9435	1356.76± 24.60	1339.90± 18.93	1320.77± 13.96	17.995	1.135	0.5315
**DL**	4.75a± 0.09	4.845a± 0.045	4.16b± 0.17	0.291	0.391	0.0002	3.97± 0.12	3.92± 0.07	4.28± 0.31	-0.151	-0.202	0.3215
**DOP**	59.74± 0.58	59.05± 0.70	59.20± 0.47	0.274	-0.420	0.6771	58.28± 0.57	58.77± 0.57	58.24± 0.22	0.024	0.511	0.7431
**liver**	5.52± 0.08	5.53± 0.08	5.54± 0.09	-0.012	0.003	0.9786	5.50± 0.07	5.57± 0.09	5.56± 0.08	-0.029	0.038	0.8021
**Kidney**	1.00± 0.01	0.99± 0.01	0.98± 0.01	0.010	0.000	0.3230	0.99± 0.01	0.99± 0.01	0.97± 0.01	0.008	0.011	0.175
**HPW**	37.97^b^± 0.302	38.82^b^± 0.33	39.12^a^± 0.44	-0.576	0.271	0.001	37.01^b^± 0.42	37.18^b^± 0.53	39.56^a^± 1.06	-1.272	-1.103	0.001

pH0 & pH24 = pH after slaughtering and 24h after slaughtering; L*^0^ & L*^24^ = Lightness (L*) after slaughtering and 24h after slaughtering; a*^0^ & a*^24^ = redness (a*) after slaughtering and 24h after slaughtering, b*^0^ & b*^24^ = yellowness (b*) after slaughtering and 24h after slaughtering; CL = cooking loss; IMF = intramuscular fat; SW = slaughter weight; HCW = hot carcass weight; CCW = commercial carcass weight; DL = Drip loss; DOP = dressing out percentage; HPW = hind part weight.

Regarding the association between the mutation of the *IRS-1* gene and different traits in the two breeds (Table 7), there was a significant association between the *IRS-1* mutation and DL in the two breeds (p < 0.008), where the GG genotype had higher DL than the TT genotype. While, in Baladi rabbits, there was a significant (p < 0.0005) reduction in IMF of GG rabbits compared to TT and GT rabbits. The results showed that the estimated dominant genetic effects were also higher than the additive genetic effects.

**Table 7:** The association analysis, by breed, of the *IRS1* c.189G>T polymorphism on carcass and meat quality traits New Zealand White and local Baladi rabbits.

	New Zealand White			Additive	Dominance	*p-value*	Local Baladi			Additive	Dominance	*p-value*
	GG	GT	TT				GG	GT	TT			
**pH0**	6.67± 0.02	6.68± 0.02	6.70± 0.03	-0.016	-0.002	0.6320	6.66± 0.01	6.65± 0.01	6.67± 0.03	-0.003	-0.012	0.7971
**pH24**	5.75± 0.02	5.77± 0.02	5.79± 0.03	-0.019	0.001	0.4156	5.75± 0.01	5.76± 0.0	5.77± 0.03	-0.014	0.000	0.5645
a* ^**0**^	4.29± 0.01	4.31± 0.01	4.31± 0.01	-0.007	0.008	0.1300	4.30± 0.01	4.29± 0.01	4.29± 0.01	0.001	0.000	0.9956
a* ^**24**^	4.81± 0.01	4.79± 0.02	4.81± 0.01	-0.001	-0.016	0.2710	4.80± 0.01	4.80± 0.01	4.82± 0.01	-0.009	-0.009	0.3817
L* ^**0**^	50.33± 0.01	50.31± 0.01	50.34± 0.01	-0.005	-0.028	0.1384	50.34± 0.01	50.32± 0.01	50.33± 0.02	0.008	-0.017	0.3293
L* ^**24**^	58.34± 0.03	58.34± 0.03	58.36± 0.06	-0.014	-0.008	0.8964	58.33± 0.04	58.37± 0.03	58.35± 0.05	-0.009	0.033	0.7223
b* ^**0**^	3.31± 0.00	3.32± 0.00	3.31± 0.01	-0.002	0.006	0.2667	3.32± 0.01	3.30± 0.02	3.32± 0.01	0.002	-0.013	0.0979
b* ^**24**^	5.52± 0.02	5.55± 0.03	5.51± 0.01	0.006	0.033	0.5859	5.53± 0.01	5.52± 0.01	5.52± 0.01	0.002	-0.006	0.7095
**CL**	27.88± 0.57	28.46± 0.55	28.61± 0.54	-0.363	0.219	0.6635	29.59± 0.60	28.71± 0.65	27.51± 0.99	1.041	0.163	0.1650
**IMF**	0.45± 0.02	0.43± 0.01	0.43± 0.02	0.008	-0.015	0.4470	0.43^a^± 0.01	0.43^a^± 0.02	0.38^b^± 0.02	0.024	0.021	0.0050
**SW**	2532.01± 19.38	2551.74± 15.45	2567.90± 23.26	-17.945	1.785	0.4703	2318.85± 33.39	2294.38± 19.497	2269.14± 20.53	24.855	0.385	0.4212
**HCW**	1561.88± 14.86	1557.84± 12.46	1580.24± 15.15	-9.180	-13.220	0.5775	1414.97± 26.28	1386.81± 16.771	1392.19± 24.37	11.390	-16.770	0.6347
**CCW**	1493.65± 14.40	1484.06± 12.32	1503.16± 14.74	-4.755	-14.345	0.6527	1359.79± 25.25	1333.52± 16.32	1331.63± 23.42	14.080	-12.190	0.5921
**DL**	4.34^b^± 0.16	4.65^ab^± 0.09	4.78^a^± 0.13	-0.222	0.0910	0.0069	3.84^b^± 0.05	3.97^ab^± 0.13	4.35^a^± 0.26	-0.256	-0.124	0.0081
**DOP**	59.65± 0.45	58.83± 0.53	59.91± 0.83	-0.130	-0.952	0.3770	58.62± 0.58	58.11± 0.40	58.65± 0.65	-0.012	-0.529	0.7177
**liver**	5.52± 0.07	5.60± 0.07	5.38± 0.12	0.070	0.153	0.1876	5.62± 0.06	5.51± 0.10	5.49± 0.07	0.067	-0.047	0.4582
**Kidney**	0.98± 0.01	0.99± 0.01	0.99± 0.01	-0.003	0.004	0.8342	1.00± 0.01	0.99± 0.01	0.98± 0.01	0.003	0.006	0.7258
**HPW**	38.63± 0.55	38.42± 0.68	39.14± 0.67	-0.256	-0.462	0.7793	37.22± 0.68	37.17± 0.51	38.60± 0.71	-0.688	-0.743	0.2269

pH0 & pH24 = pH after slaughtering and 24h after slaughtering; L*^0^ & L*^24^ = Lightness (L*) after slaughtering and 24h after slaughtering; a*^0^ & a*^24^ = redness (a*) after slaughtering and 24h after slaughtering, b*^0^ & b*^24^ = yellowness (b*) after slaughtering and 24h after slaughtering; CL = cooking loss; IMF = intramuscular fat; SW = slaughter weight; HCW = hot carcass weight; CCW = commercial carcass weight; DL = Drip loss; DOP = dressing out percentage; HPW = hind part weight.

## 4. Discussion

Association analysis between traits and polymorphisms in animals is important because it can help to identify genetic variants that are associated with economic traits [44]. This can provide insight into the underlying genetic mechanisms that contribute to these traits. Also, this analysis has a high economic value, as its results can be implemented in breeding programs to improve production and animal revenue. However, very limited studies addressed the association of polymorphisms of different genes with quantitative traits in rabbits [10, 33]. Therefore, the current study evaluated the association of four SNPs located in four different genes with carcass and meat quality traits in two rabbit breeds.

The evaluation of the studied traits revealed significant differences between the two breeds in four carcass traits, including slaughter weight (SW), hot carcass weight (HCW), commercial carcass weight (CCW), and drip loss (DL). The difference in slaughter weight may be attributed mainly to the difference in growth rates between the two breeds. The differences in finishing weight (12 weeks of age) between NZW and Egyptian Baladi rabbits were previously reported [37]. These differences are usually extendable and lead to differences in slaughter and hot carcass weights. Moreover, important differences between different genetic groups in slaughter, carcass and meat quality traits were reported [3]. Finishing body weight is considered the most important live body variable in explaining variation in carcass traits [45]. Nonetheless, Hernández et al. [46] reported that live body weight measurements are not good indicators for carcass traits.

The genotypic frequencies obtained for all SNPs were consistent with HW equilibrium indicating that the two breeds used are under circumstances of random mating. Also, this may be because the rabbits used for the current work were gathered from different source, and all individuals were genetically unrelated. Although the allelic frequency of the c.-78C>T of the *GH* gene was not greatly different between the NEZ and LB rabbits, the predominant allele was not the same in the two breeds, which implies the existence of breed effect for this locus. Ramadan et al. [15] reported frequencies of 0.63 and 0.37 for alleles C and T when they genotyped the same mutation in Egyptian Gabali rabbits. A higher frequency for allele C was obtained in Termond White (0.68) and in Belgian Giant Grey (BGG, 0.85) rabbits [20], however, the allele T was predominant (0.72) in the NZW × BGG crossbred. The same difference between the breeds in the predominant allele was observed for the *IGF2* c.156+61delA mutation. The deletion mutation frequency on the same locus in Gabali rabbits was as high as 0.62 [15]. Fontanesi et al. [22] genotyped the same mutation and reported a frequency for the del allele ranged from 0.00 (in Belgian Hare and Mini Loop rabbits) to 0.22 (in NZW rabbits). The *FTO* mutation was genotyped using re-sequencing technique in different rabbit breeds, and the frequency of the G allele was high in all breeds and ranged between 0.81 in NZW rabbits and 0.99 in Tianfu black rabbits [28]. In the current study, the allele G was predominant in the two breeds with an average frequency of 0.57. The same was observed for IRS1 c.189G>T SNP as the frequency of allele T was higher than allele G with an average of 0.55. Similar trend for the frequency in NZW rabbits was obtained previously for alleles G and T with frequencies of 0.61 and 0.39, respectively [35]. Helal et al. [9] reported frequencies for allele G of 0.54, 0.55, and 0.59 in NZW, Baladi Red, and V-line rabbits, respectively.

In the current study, the four studied genes have important roles in controlling growth and muscle development. The *GH* gene plays a very important role in postnatal growth and muscle deposition [17]. It has also another function in lipid metabolism. The product of the *IGF-2* gene is insulin-like growth factor-II (IGF-II), which belongs to the same family as IGF-I [47]. Furthermore, the growth hormone is an effector of the *IGF-II* promoter-specific transcription [48]. The *FTO* gene is not only an obesity-susceptibility gene, it also affects growth, particularly postnatal growth [49]. Moreover, GH has a function in the modulation of the relationships between the *FTO* gene and body mass index [50]. Also, the product of the *IRS-1* gene is the IRS1 protein that has a role in the signal transudation of both *GH* and *IGF-2* genes [51]. Therefore, we combined those four genes in one study as we were expecting similar association results due to the strong relationships among them.

The polymorphisms of the *GH* gene were previously associated with carcass traits, the same SNP (c.-78C>T) genotyped in the current study was previously associated with slaughter weight in Belgian Giant Gray (BGG) rabbits, and was also associated with HCW, HPW, and meat in hind parts in BGG × NZW crossbred rabbits [20]. However, the results of the current study did reveal any association with carcass traits except for HPW. This GH mutation was also associated with growth traits. Fontanesi et al. [17] reported an association between c.-78C>T SNP and finishing weight (body weight at 70 days of age) in commercial rabbits. It was also associated with birth weight and body weight at 35 days of age in BGG rabbits [20]. In Egypt, the association of the same SNP with body weights at 28, 42, and 56 days of age in the APRI and Moshtohor rabbits was reported [19], and with body weights at 28, 56, 70, and 84 days of age in Moshtohor and V-line rabbits [18]. El-Sabrout and Aggag [52] also genotyped the *GH* gene in V-line and Alexandria-line rabbits, but they did not detect any mutations.

The relationship between GH and IGFs is well documented, where GH had anti-insulin activities [53]. For this reason, the association results for the SNP of the *IGF-2* gene with carcass traits were likely to be similar to that of the *GH* gene SNP as expected, as the association was only observed in the case of HPW. Although there is a strong relationship between *GH* and *IGF-2*, as previously stated, there is a limited number of studies that explored the polymorphism of *IGF2* in rabbits. No previous study addressed the association of *IGF2* mutations with carcass or meat quality traits in rabbits. Fontanesi et al. [22] studied the association between the same mutation (c.156+61delA) and growth traits in commercial rabbits and reported a significant association with finishing weight (body weight at 70 days of age). Also, Ramadan et al. [15] reported a significant association between the Del/Del genotype of the same mutation with body weights at 28, 56, and 84 days of age, and it was also associated with daily weight gain from 28 to 56 days of age, and from 56 to 84 days of age. The *IGF2* gene polymorphisms were associated with carcass and meat quality traits in other animals such as rib-eye in cattle [54], marbling, tenderness, pH24 of meat quality in Qinchuan cattle [55], inter-muscular fat content in pork [56], and carcass weight at 17 weeks of age in chickens [57].

The *FTO* is another important gene that was not deeply subjected to association studies in rabbits. Results of the current study suggested the association between the SNP of the *FTO* gene with CL, HPW, IMF, and L*^0^. Zhang et al. [28] genotyped another SNP (c.479A>G) located in the same gene and reported a significant association with IMF in Ira rabbits. However, the SNP c.499G>A was associated with growth traits (body weights at 35, 70, and 84 days of age) in NZW, Champagne, and Ira rabbits, but not with carcass or meat quality traits [28]. Intramuscular fat is associated with the amount of red muscle fiber [58]. The *FTO* gene is expressed in adipose tissues and the skeletal muscles, and has a critical function in regulating appetite and energy metabolism [59]. The pathway of FTO is targeting many other genes, among them is the adipocyte plasma membrane associated protein (APMAP) gene, which may be the major effector of IMF content [60]. Therefore, the *FTO* gene can be used as a candidate gene for IMF content in rabbits.

The *IRS-1* protein has important roles in signal transduction of both *IGF2* and *GH* genes and has a contributory role in controlling body weight [61]. The genotyped mutation of the *IRS-1* gene is located between two tyrosine phosphorylation sites (Tyr46 and Tyr87) and was reported to affect the body weights at 70 and 84 days of age as well as average daily gain in rabbits [35]. The association between *FTO* and *IRS-1* genes was studied [35] and it revealed that SNP combinations of both genes regulate body weights in rabbits. Recently, significant associations between the same studied SNP (c.189G>T) of the *IRS-1* gene and some growth traits including body weight at 42 days of age in NZW, V-line, and Baladi Red rabbits were reported [9]. Nevertheless, there is no previous studies addressing the association of *IRS-1* gene polymorphism with carcass and meat quality traits in rabbits.

The associations between the polymorphism of SNPs and phenotypic traits are essential for the genetic improvement of rabbit breeds and the development of new lines, which can be achieved using a marker-assisted selection approach. Such an approach is important particularly when the breeding program aims at improving traits that are difficult to be measured on live animals or traits with low levels of variation [7]. Carcass and meat quality traits are good examples of traits that are difficult to be improved using mass selection due to expensive measurement and the necessity to slaughter animals [62]. Exploring the associations between polymorphism of different genes and those traits offer great opportunities for accelerating the improvement of carcass and meat quality traits in rabbits [63]. The results of the current study did not revealing associations between the studied SNPs and meat quality traits except for L*^0^, L*^24^, and IMF. However, there were associations with carcass traits including SW, HCW, CCW, CL, and HPW. The limited association results with meat quality may be attributed to many reasons such as the small variability at those loci in rabbits, and it may be attributed to the low heritability estimates for many meat quality traits compared with carcass and growth traits [64]. Furthermore, measuring the quality of meat is affected by different environmental factors including feeding, health, and cellular metabolism, as well as the quality of the sensors used for measuring the different traits [65]. Overall, there is limited research on the relationship between the studied genes and meat quality in rabbits, and therefore more research is needed to fully understand the mechanisms underlying this relationships among them and to determine the practical implications for rabbit meat production.

## 5. Conclusion

The results revealed a significant effect for *GH* genotypes on meat lightness after slaughter and hind-part weight. While, the *IGF2* mutation significantly affected slaughter, hot carcass, commercial carcass, and hind-part weights. The *FTO* SNP was associated with cooking loss and intramuscular fat weight, and the *IRS-1* SNP was significantly associated with drip loss and intramuscular fat. Novel associations of *FTO* c.499G> A, and *IRS-1* c.189G>T SNPs with intramuscular fat were detected and they have not been addressed before. Collectively, the results underlined the role of SNPs in candidate genes as sources of variability that can affect quantity and quality in rabbit meat production that are under polygenic control and highlighted the need for more research for validating the current findings and identifying more candidate/causative mutations.

## Supporting information

S1 Data (XLSX)
